# A nomogram based on the preoperative neutrophil-to-lymphocyte ratio to distinguish sarcomatoid renal cell carcinoma from clear cell renal cell carcinoma

**DOI:** 10.3389/fonc.2023.1218280

**Published:** 2023-09-22

**Authors:** Yijian Wu, Tienan Qi, Xin Qin, Zhongwei Zhao, Jianguo Zheng, Qinglong Du, Nengwang Yu

**Affiliations:** ^1^ Department of Urology, Qilu Hospital of Shandong University, Jinan, Shandong, China; ^2^ Cheeloo College of Medicine, Shandong University, Jinan, Shandong, China

**Keywords:** sarcomatoid renal cell carcinoma, clear cell renal cell carcinoma, neutrophil-to-lymphocyte ratio, nomogram, distinguish

## Abstract

**Objective:**

Our study aimed to assess the predictive value of the preoperative neutrophil-to-lymphocyte ratio(NLR) in distinguishing sarcomatoid renal cell carcinoma (SRCC) from clear cell renal cell carcinoma(CCRCC) and to developing a nomogram based on the preoperative NLR and other factors to distinguish SRCC from CCRCC.

**Materials and methods:**

The database involved 280 patients, including 46 SRCC and 234 CCRCC. logistic analysis was conducted to select the variables associated with identifying SRCC preoperatively, and subgroup analysis was used to further validate the ability of NLR with preoperative identification of SRCC.In addition, The data were randomly separated into a training cohort(n=195) and a validation cohort(n=85). And an NLR-based nomogram was plotted based on the logistic analysis results. The nomogram was evaluated according to its discrimination, consistency, and clinical benefits.

**Results:**

Multivariate analysis indicated that NLR, flank pain, tumor size, and total cholesterol(TC) were independent risk factors for identifying SRCC. The results of subgroup analysis showed that higher NLR was associated with a higher probability of SRCC in most subgroups. The area under the curve(AUC) of the training and validation cohorts were 0.801 and 0.738, respectively. The results of the calibration curve show high consistency between predicted and actual results. Decision Curve Analysis(DCA) showed clinical intervention based on the model was beneficial over most of the threshold risk range.

**Conclusion:**

NLR is a potential indicator for preoperative differentiation of SRCC and CCRCC, and the predictive model constructed based on NLR has a good predictive ability. The new model could provide suggestions for the early identification of SRCC.

## Background

Renal cell carcinoma (RCC) is reported to account for about 4% of all human malignancies. About 180,000 people die from RCC worldwide yearly, with more than 400,000 new cases diagnosed in 2018 (1, 2). RCC includes several pathological types, the majority of which, 70% to 80% of cases, are clear cell carcinoma (3, 4). Sarcomatoid renal cell carcinoma(SRCC) refers to RCC that occurs with sarcomatoid differentiation and can occur in all subtypes of RCC (5). SRCC is uncommon, with an average incidence of 5% to 7% (6), but it is highly aggressive, accounting for about 10% to 20% of advanced RCC (7, 8). Compared to CCRCC, SRCC has a poorer prognosis (7). The International Society of Urological Pathology (ISUP) classifies RCC as grade IV when sarcomatoid differentiation occurs (9). The treatment strategy for SRCC is different from that for CCRCC. Shuch et al. reported that there might be no clear survival benefit of cytoreductive nephrectomy in patients with SRCC and recommended that patients with preoperatively determined SRCC receive upfront systemic therapy (10). Crispen et al. recommended lymph node dissection during radical nephrectomy for SRCC (11). Unfortunately, there is currently no reliable method to differentiate between SRCC and CCRCC preoperatively. Previous studies have shown that risk factors such as a large necrotic area (12), a large tumor size (12, 13), and an increased number and volume of neovascularization around tumors (12, 13) are all associated with a preoperative diagnosis of SRCC. However, most studies focused only on imaging features and ignored hematological indicators and clinical factors of patients. Therefore, a new predictive model is needed to identify SRCC preoperatively.

The neutrophil-to-lymphocyte ratio(NLR) as an inflammatory marker represents two responses of the body to cancer. Neutrophils reveal the systemic inflammatory response, and lymphocytes reflect the immune profile of the body. NLR has been frequently used to distinguish between benign and malignant tumors and categorize the tumor aggressiveness level (14–16). However, no previous studies have used NLR as a predictor for preoperative identification of SRCC and CCRCC, and the relationship between them has not been validated.

In this study, we aimed to assess whether NLR is a potential preoperative predictor of SRCC and to develop a predictive model for preoperative differentiation between SRCC and CCRCC by including the patient’s NLR.

## Materials and methods

### Data collection

From 2013 to 2019, the clinical data from patients treated with radical or partial nephrectomy and were pathologically diagnosed with SRCC or CCRCC were retrospectively analyzed. The following were the inclusion requirements: (1) No history of cancer; (2) No hematologic diseases to avoid interference with hematologic indicators; (3) Complete postoperative pathology results; (4) Complete clinical data; (5) No evidence of extrarenal metastasis. (6) Tumor size>4 cm.

### Risk factors

We collected the following clinical information from the patient’s medical charts: age, sex, symptoms (flank pain, haematuria, proteinuria), past medical history(hypertension, diabetes), types of nephrectomy, pathological features, tumor size, and preoperative peripheral blood indicators(white blood cell count, neutrophils count, lymphocyte count, cholesterol, blood urea nitrogen, creatinine, triglycerides). NLR is obtained by calculating(neutrophils count/lymphocyte count). The patient’s past medical history and symptoms were obtained from the admission records. All pathological features were according to the postoperative pathology report. Tumor size depends on the longest tumor diameter. A sample of peripheral blood is obtained within 14 days before surgery.

### Statistical analysis

The continuous data were described as mean with standard deviation (SD) and median with range and tested with the Mann-Whitney U test and student t-tests. The categorical data were described as frequencies and tested with the χ2 test. Use ROC curves to determine the cut-off values of continuous variables. Correlation analysis used the Spearman rank correlation coefficient. We used logistic analyses to screen for predictive variables and incorporate those variables that were statistically significant into the predictive model. The participants are assigned into training and validation cohorts in a 7:3 ratio using the createDataPartition function in the “Caret” package in R (version 3.4.0), with no statistically significant changes in any of the characteristics between the two cohorts. The model is presented as a dynamic nomogram, and summing each patient’s score on each predictive factor allows the calculation of the patient’s total score and calculates the patient’s risk of the outcome event. To evaluate the model’s capacity for prediction, we employed calibration curves and receiver operating characteristic (ROC) analysis. The net benefits of clinical interventions based on the model have been evaluated using decision curve analysis (DCA).

Statistical analyses were performed using SPSS software (version 26.0, US), R software (version 3.4.0, US), and GraphPad Prism(9.0). P<0.05 indicates a statistically significant difference.

## Results

### Patient characteristics

After the screening, 280 patients were enrolled (**Table 1**), including 234 CCRCC and 46 SRCC. There were 187 males and 93 females. The median age of patients was 57 years (25~85), and the median tumor size was 6cm (4.2~17.5cm). 57 patients (20.4%) had flank pain on admission and 245 patients (87.5%) underwent radical nephrectomy. Median NLR and total cholesterol (TC) were 2.27 (0.65~16.71) and 4.38 (1.03~6.75), respectively. The mean NLR for the SRCC cohort was 3.36 ± 1.85, higher than the 2.43 ± 1.52 for the CCRCC cohort, and we plotted ROC curves based on NLR levels for all patients, which showed an AUC value of 0.704 (**Supplementary Figure 1**).

**Table 1:** Baseline characteristics of 280 RCC patients in this study.

	Overall (n=280)	CCRCC (n=234)	SRCC (n=46)
Age (years)			
Mean (SD)	57.0 (10.6)	56.5 (10.4)	59.7 (11.3)
Median [Min,MAX]	57.0 [25,85]	57.0 [31,85]	62.0 [28,76]
Sex			
Male	187 (66.3%)	157 (67.1%)	30 (65.2%)
Female	93 (33.7%)	77 (32.9%)	16 (34.8%)
Hypertension			
Yes	102 (36.4%)	86 (36.8%)	16 (34.8%)
No	178 (63.6%)	148 (63.2%)	30 (65.2%)
Diabetes			
Yes	36 (12.9%)	30 (12.8%)	6 (13.0%)
No	244 (87.1%)	204 (87.2%)	40 (87.0%)
Flank Pain			
Yes	57 (20.4%)	40 (17.1%)	17 (37.0%)
No	223 (79.6%)	194 (82.9%)	29 (63.0%)
Haematuria			
Yes	47 (16.8%)	34 (14.5%)	13 (28.3%)
No	233 (83.2%)	200 (85.5%)	33 (71.7%)
Preoperative proteinuria			
Yes	40 (14.3%)	32 (13.7%)	8 (17.4%)
No	240 (85.7%)	202 (86.3%)	38 (82.6%)
T stage			
≤II	216 (77.1%)	190 (81.2%)	26 (56.5%)
≥III	64 (22.9%)	44 (18.8%)	20 (43.5%)
Size (cm)			
Mean (SD)	6.6 (2.1)	6.3 (1.8)	7.9 (2.9)
Median [Min,MAX]	6.0 [4.2,17.5]	6.0 [4.2,14]	7.0 [4.2,17.5]
White blood cell count			
Mean (SD)	6.32 (1.73)	6.19 (1.71)	6.96 (1.71)
Median [Min,MAX]	6.07 [2.60,14.78]	5.90 [2.60,14.78]	6.71 [2.85,10.85]
Neutrophils count (10^9^/L)			
Mean (SD)	4.00 (1.50)	3.85 (1.46)	4.70 (1.50)
Median [Min,MAX]	3.82 [0.96,12.87]	3.62 [0.96,12.87]	4.81 [1.68,7.97]
Lymphocyte count (10^9^/L)			
Mean (SD)	1.71 (0.52)	1.74 (0.52)	1.57 (0.50)
Median [Min,MAX]	1.66 [0.53,3.36]	1.68 [0.68,3.36]	1.49 [0.53,2.70]
NLR			
Mean (SD)	2.58 (1.62)	2.43 (1.52)	3.36 (1.85)
Median [Min,MAX]	2.27 [0.65,16.71]	2.12 [0.65,16.71]	2.90 [0.79,10.53]
TC (mmol/L)			
Mean (SD)	4.39 [0.88]	4.47 (0.88)	3.98 (0.77)
Median [Min,MAX]	4.38 [1.03,6.75]	4.45 [1.03,6.75]	3.88 [2.3,6.05]
BUN (mmol/L)			
Mean (SD)	4.96 (1.35)	5.00 (1.32)	4.74 (1.45)
Median [Min,MAX]	4.80 [2.25,9.8]	4.81 [2.30,9.34]	4.37 [2.25,9.80]
Cr (u mol)			
Mean (SD)	71.4 (16.4)	71.3 (16.5)	71.8 (16.1)
Median [Min,MAX]	69 [38,129]	69 [38,129]	70 [38,103]
TG (mmol/L)			
Mean (SD)	1.29 (0.60)	1.34 (0.61)	1.06 (0.51)
Median [Min,MAX]	1.14 [0.32,3.62]	1.20 [0.32,3.62]	0.91 [0.49,2.76]
Type of nephrectomy			
Partial	35 (12.5%)	32 (13.7%)	3 (6.5%)
Radical	245 (87.5%)	202 (86.3%)	43 (93.5%)

CCRCC, clear cell renal cell carcinoma; SRCC, sarcomatoid renal cell carcinoma; NLR, neutrophil-to-lymphocyte ratio; TC, total cholesterol; BUN, blood urea nitrogen; Cr, creatinine; TG: triglycerides.

### Univariable and multivariable analyses of variables

Univariate analysis suggested that flank pain (P=0.003), tumor size (P=0.001), NLR (P=0.004), TC (P=0.001), T stage (P=0.001), and haematuria (P=0.026) were statistically significant risk factors. Multivariate analysis showed that flank pain (HR: 2.84, 95% CI: 1.30~6.22, P=0.009), tumor size (HR: 1.25, 95% CI: 1.06~1.47, P=0.009), NLR (HR: 1.27. 95% CI: 1.06~1.52, P=0.008) and TC (HR: 0.52, 95% CI: 0.34~0.79, P=0.003) were independent risk factors for predicting SRCC. Haematuria (HR: 1.54, 95% CI: 0.54~4.43, P=0.420) and T stage (HR: 2.20, 95% CI: 1.00~4.83, P=0.051) was not an independent risk factor (**Table 2**).

**Table 2:** Univariate and multivariate logistic analyses for the risk of SRCC.

Parameters	Univariate analysis HR (95%CI)	P value	Multivariate analysis HR (95%CI)	P value
Age	1.03 (1.00~1.06)	0.064		
Sex	0.92 (0.47~1.79)	0.805		
Hypertension	0.92 (0.47~1.78)	0.800		
Diabetes	1.02 (0.40~2.61)	0.967		
Flank Pain	2.84 (1.43~5.66)	0.003	2.84 (1.30~6.22)	0.009
Haematuria	2.32 (1.11~4.85)	0.026	1.42 (0.60~3.36)	0.431
Proteinuria	1.33 (0.57~3.11)	0.511		
T stage	3.32 (1.70~6.48)	0.001	2.20 (1.00~4.83)	0.051
Size	1.36 (1.18~1.56)	0.001	1.25 (1.06~1.47)	0.009
NLR	1.32 (1.09~1.60)	0.004	1.27 (1.06~1.52)	0.008
TC	0.51 (0.35~0.75)	0.001	0.52 (0.34~0.79)	0.003
BUN	0.86 (0.67~1.10)	0.239		
Cr	1.00 (0.99~1.02)	0.843		
TG	1.04 (0.97~1.12)	0.233		

NLR, neutrophil-to-lymphocyte ratio; TC, total cholesterol; BUN, blood urea nitrogen; Cr, creatinine; TG, triglycerides; HR, hazard ratio; CI, confidence interval.

### Relationship between NLR and clinical characteristics

**Figure 1:**
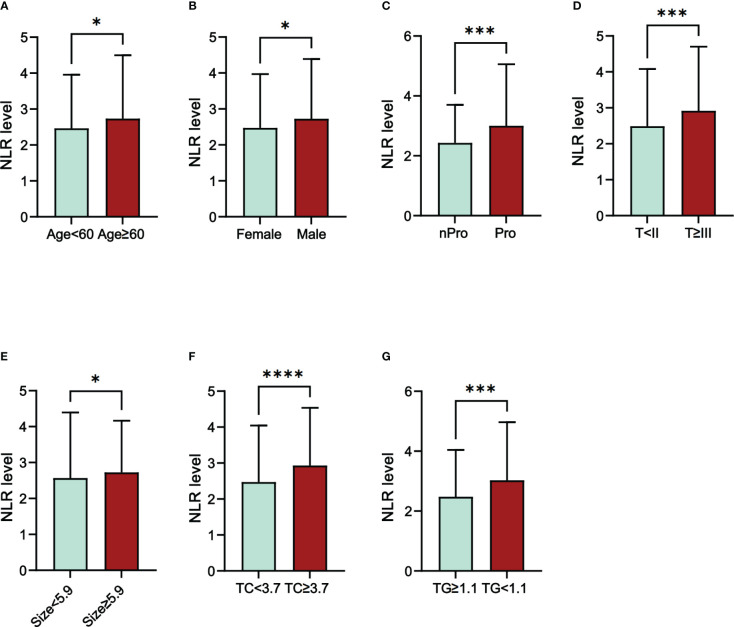
The differences in NLR level in different groups of RCC patients. **(A)** Age; **(B)** Sex; **(C)** Proteinuria; **(D)** T stage; **(E)** Size; **(F)** TC; **(G)** TG; Abbreviations: NLR, neutrophil-to-lymphocyte ratio; Pro, Proteinuria; nPro, no Proteinuria; TC, total cholesterol; TG, triglycerides; *P<005; ***P<0.001; ****P<0.0001.

We also investigated the correlation between NLR and the clinical characteristics of RCC patients (**Table 3**). Cut-off values for continuous variables were determined using ROC curves: Age (60 y); tumor size (5.9 cm); TC (3.7 mmol/l); BUN (5.2mmol/l); Cr (82.5mmol/l); TG (1.1mmol/L). The results showed that NLR level correlated with age (P=0.040), sex (P=0.003), proteinuria (P=0.003), T stage (P=0.001), tumor size (P=0.013), TC (P<0.001), and TG (P<0.001) (**Figure 1**). We further assessed the correlation between NLR and age, tumor size, TC, and TG using linear correlation analysis. The results showed that NLR did not correlate with age (P=0.08) and TG (P=0.07) and correlated weakly with tumor size (r=0.23) and TC (r=0.24) (**Supplementary Figure 2**).

**Table 3:** Relationship between NLR and clinical characteristics.

Clinical characteristics	n (%)	NLR (Mean ± SD)	P-value
Age (years)			0.040
≥60	121 (43.2%)	2.74 (1.76)	
<60	159 (56.8%)	2.47 (1.49)	
Sex			0.003
Male	187 (66.8%)	2.73 (1.66)	
Female	93 (33.2%)	2.29 (1.48)	
Hypertension			0.407
Yes	102 (36.4%)	2.56 (1.86)	
No	178 (63.6%)	2.60 (1.46)	
Diabetes			0.753
Yes	36 (12.9%)	2.53 (1.16)	
No	244 (87.1%)	2.59 (1.67)	
Flank Pain			0.776
Yes	57 (20.4%)	2.49 (1.04)	
No	223 (79.6%)	2.61 (1.73)	
Haematuria			0.075
Yes	47 (19.4%)	2.70 (1.07)	
No	223 (80.6%)	2.56 (1.71)	
Proteinuria			0.003
Yes	40 (16.9%)	3.47 (2.80)	
No	240 (83.1%)	2.43 (1.27)	
T stage			0.001
II	216 (77.1%)	2.48 (1.64)	
≥III	64 (22.9%)	2.93 (1.51)	
Size (cm)			0.013
≥5.9	163 (58.2%)	2.67 (1.35)	
<5.9	117 (41.8%)	2.47 (1.93)	
TC (mmol/L)			<0.001
≥3.7	218 (77.9%)	2.43 (1.56)	
<3.7	62 (22.1%)	3.13 (1.70)	
BUN (mmol/L)			0.874
≥5.2	106 (37.9%)	2.78 (2.26)	
<5.2	174 (62.1%)	2.46 (1.03)	
Cr (u mol)			0.059
≥82.5	66 (23.6%)	2.90 (2.12)	
<82.5	214 (76.4%)	2.48 (1.42)	
TG (mmol/L)			<0.001
≥1.1	153 (54.6%)	2.32 (1.29)	
<1.1	127 (45.3%)	2.90 (1.90)	

NLR, neutrophil-to-lymphocyte ratio; TC, total cholesterol; BUN, blood urea nitrogen; Cr, creatinine; TG, triglycerides.

### Subgroup analysis

**Figure 2:**
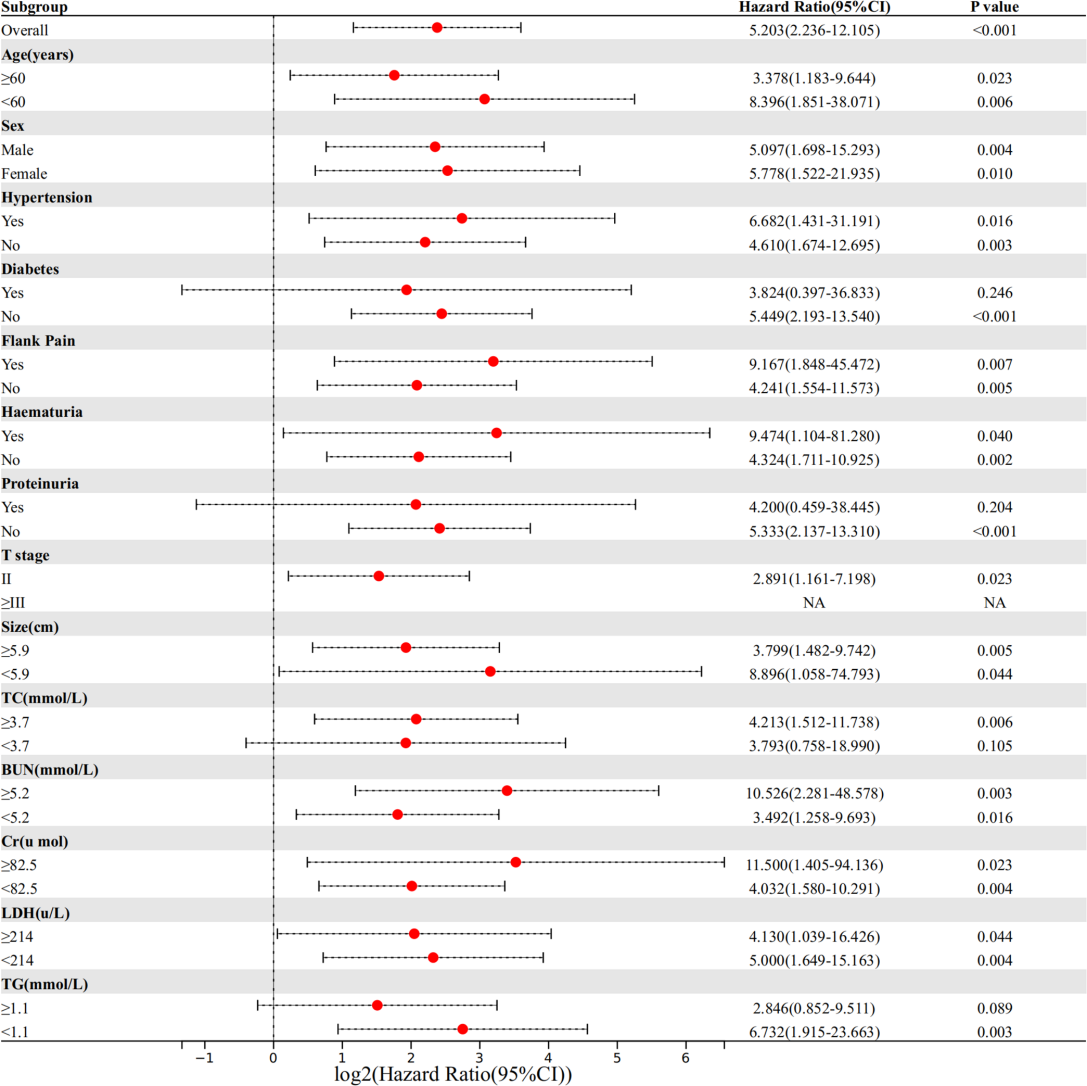
Comparison of the difference in risk of SRCC between the high NLR group and low NLR group in different subgroups.

We divided NLR into a high NLR group and a low NLR group according to the cut-off value (2.1) of NLR. and performed subgroup analysis (**Figure 2**). The results showed that in most subgroups, the high NLR group was associated with a higher probability of SRCC.

### Dynamic nomogram development

**Figure 3:**
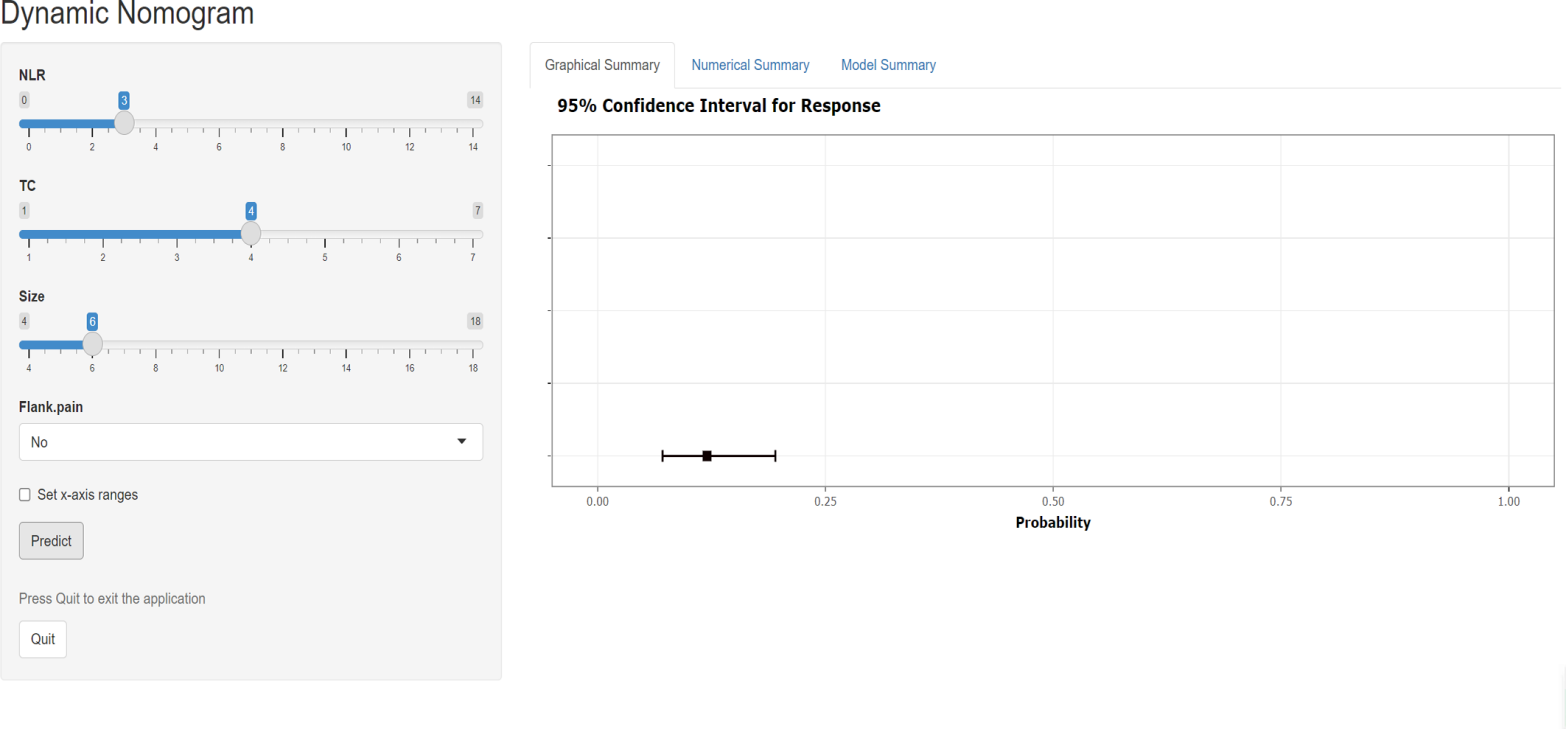
Screenshot of the dynamic nomogram for distinguishing sarcomatoid renal cell carcinoma from clear cell renal cell carcinoma. The figure shows the probability of predicting SRCC with an input NLR of 3, TC of 4, size of 6, and no flank pain. NLR neutrophil-to-lymphocyte ratio, TC total cholesterol.

The training and validation cohorts had no statistical differences in clinical factors, hematological indicators, or pathological features after random grouping, according to 7:3 (**Supplementary Table 1**). In the training group, both univariate and multivariate logistic analyses for the four predictors mentioned above were statistically significant (**Supplementary Table 2**). We incorporated the above four predictors into the predictive model and generated the dynamic nomogram(https://nomogramsrcc.shinyapps.io/DynNomappSrcc/) (**Figure 3**). In the dynamic nomogram, each predictor value corresponds to a score, and the four scores for each patient are summed to obtain the total score. The risk of SRCC can be estimated by finding the risk rate corresponding to the total score.

### Nomogram validation

**Figure 4:**
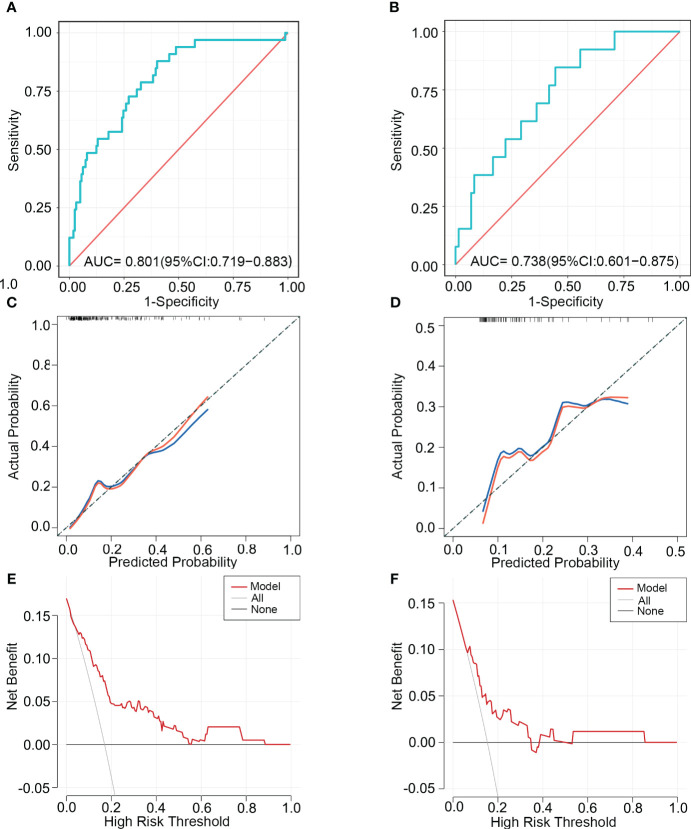
The ability of the model to discriminate SRCC was validated using ROC, calibration, and DCA curves. ROC curves **(A)**, calibration curves **(C)**, and DCA curves **(E)** of the training cohort. ROC curves **(B)**, calibration curves **(D)**, and DCA curves **(F)** of the validation cohort. ROC, receiver operating characteristic; AUC, area under the curve; DCA, Decision Curve Analysis.

We used ROC, calibration, and DCA curves to assess the discrimination, consistency, and clinical benefit of the model, respectively. The ROC curve results showed an AUC value of 0.801 for the training cohort and 0.738 for the validation cohort (**Figures 4A, B**). We also compared the AUC values of the nomogram with the AUC values of each predictive factor(**Supplementary Table 3**). The findings demonstrated that the nomogram’s AUC values were higher than any single predictor’s. The above results suggested that the predictive model had good discrimination. The calibration curve results showed that the curves of the two cohorts had a high overlap with the diagonal line, indicating that the model’s projected probability and the actual probability agreed rather well (**Figures 4C, D**). The DCA curve results suggested a net clinical benefit for clinical decisions based on the predictive model for most of the threshold probability range in the training and validation cohorts (**Figures 4E, F**).

### Stratifying risk based on nomogram

**Figure 5:**
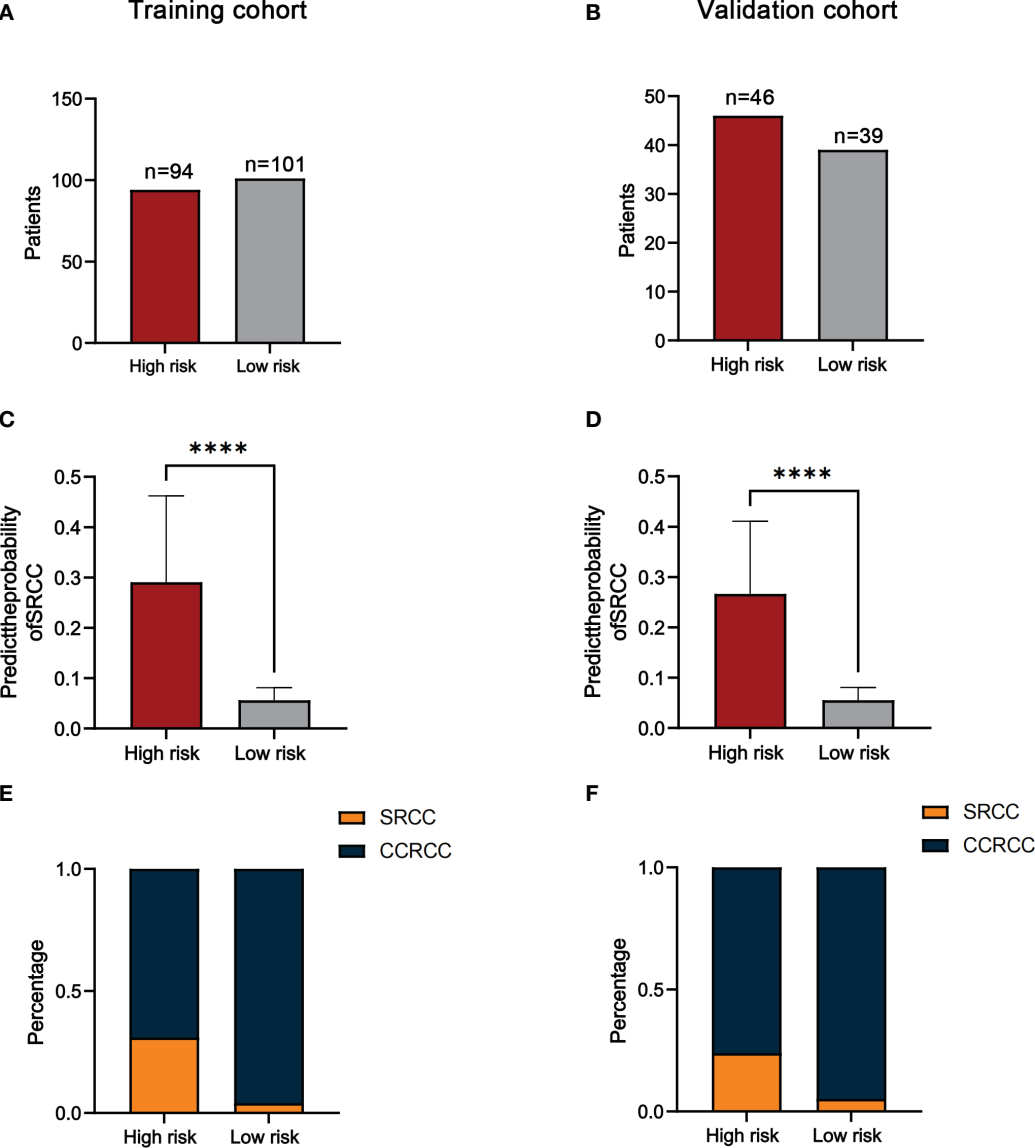
The number of patients in the high-risk and low-risk groups in the training **(A)** and validation cohorts **(B)**. Comparison of the probability of predicting SRCC in high-risk and low-risk groups in the training **(C)** and validation cohorts **(D)**. The proportion of SRCC distribution in high-risk and low-risk groups for the training **(E)** and validation cohorts **(F)**. ****P<0.0001.

We used ROC curves to determine the cut-off value (74.9) of the predicted score for the training cohort and divided the training and validation cohorts into a high-risk group (≥74.9) and a low-risk group (<74.9) based on this value. The results showed that the number of high-risk and low-risk patients in the training cohort was 94 and 101 (**Figure 5A**). The number of high-risk and low-risk patients in the validation cohort was 46 and 39 (**Figure 5B**). The median predicted probabilities for the high-risk and low-risk groups in the training cohort were 22.7% and 5.8%(P<0.0001) (**Figure 5C**). The median predicted probabilities for the high-risk and low-risk groups in the validation cohort were 23.7% and 4.6%(P<0.0001) (**Figure 5D**). The number of SRCC patients in the high-risk and low-risk groups in the training cohort was 29 (30.9%) and 4 (4.0%)(P<0.001) (**Figure 5E**). The number of SRCC patients in the validation cohort in the high-risk and low-risk groups was 11 (23.9%) and 2 (5.1%) (P=0.016) (**Figure 5F**).

**Figure 6:**
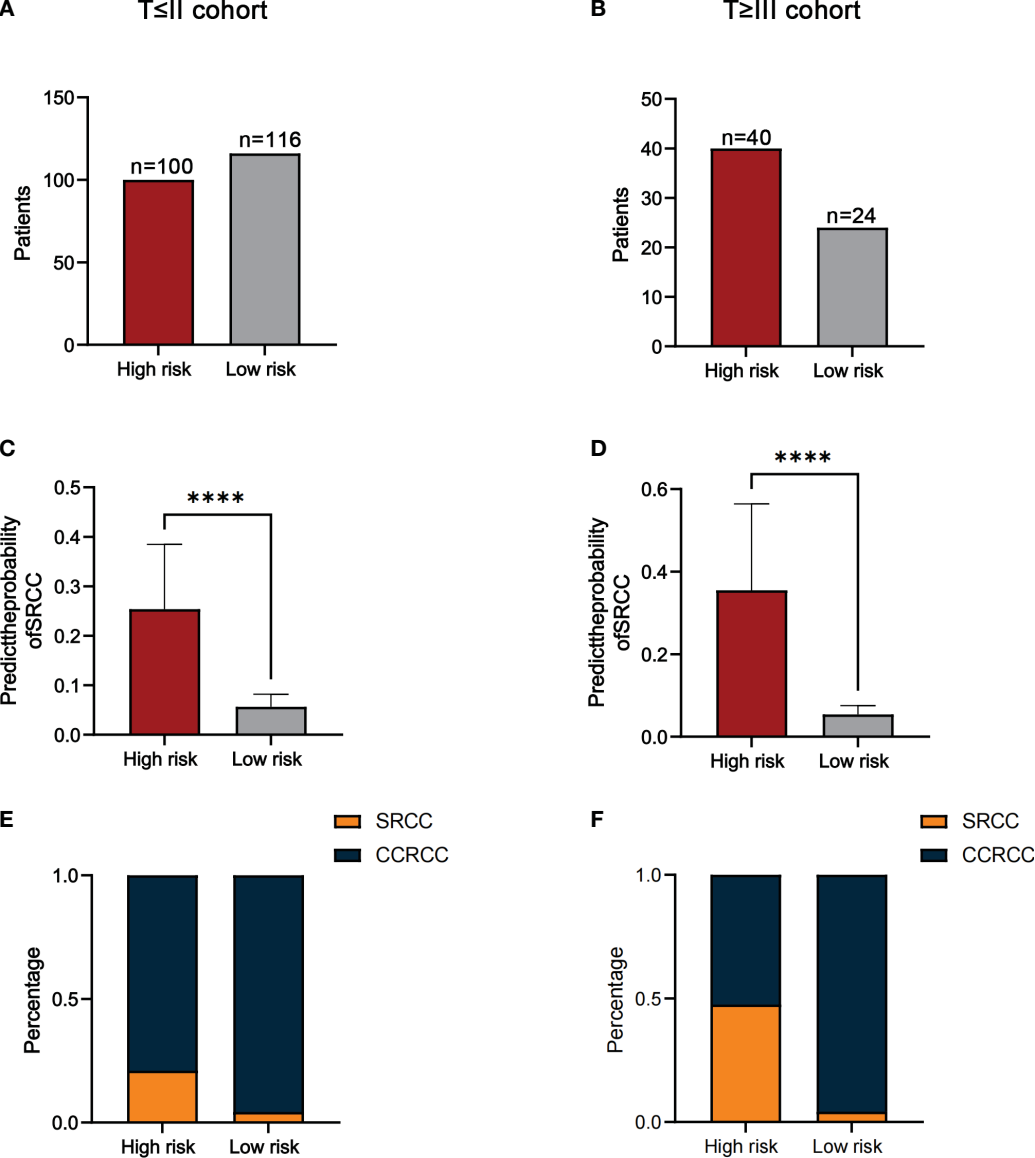
The number of patients in the high-risk and low-risk groups in the T≤II **(A)** and T≥III cohorts **(B)**. Comparison of the probability of predicting SRCC in high-risk and low-risk groups in the T≤II **(C)** and T≥III cohorts **(D)**. The proportion of SRCC distribution in high-risk and low-risk groups for the T≤II **(E)** and T≥III cohorts **(F)**. ****P<0.0001.

In addition, since SRCC and CCRCC were considered to have different disease progression, we divided patients into two subgroups(T≤II and T≥III) based on T-staging and further validated the predictive ability of the model. The results showed that the number of high-risk and low-risk patients in the T≤II subgroup was 100 and 116 (**Figure 6A**). The number of high-risk and low-risk patients in the T≥III subgroup was 40 and 24(**Figure 6B**). The median predicted probabilities for the high-risk and low-risk groups in the T≤II subgroup were 22.9% and 5.8%(P<0.0001) (**Figure 6C**). The median predicted probabilities for the high-risk and low-risk groups in the T≥III subgroup were 30.4% and 5.4%(P<0.0001) (**Figure 6D**). The number of SRCC patients in T≤II subgroup in the high-risk and low-risk groups was 21 (21.0%) and 5 (4.3%)(P<0.001) (**Figure 6E**). The number of SRCC patients in the T≥III subgroup in the high-risk and low-risk groups was 19 (47.5%) and 1 (4.2%)(P<0.001) (**Figure 6F**).

In conclusion, the results suggest that patients in the high-risk group are more likely to develop SRCC than low-risk group patients.

## Discussion

SRCC is the RCC that occurs with sarcomatoid dedifferentiation and can be found in all subtypes (5). SRCC progresses rapidly, with a median survival of 6-13 months (17), and has the worst prognosis of RCC (18). SRCC is treated differently from CCRCC, and preoperative systemic therapy and intraoperative lymph node dissection can improve the prognosis of patients (7, 10, 11). A reliable predictive model is needed to identify SRCC in a timely manner. In this study, we included for the first time preoperative hematological indicators and clinical factors in patients and constructed the first predictive model (including NLR, flank pain, tumor size, and TC) to distinguish preoperatively between SRCC and CCRCC based on patients’ preoperative NLR levels. We also investigated the correlation between NLR level and clinical characteristics of RCC patients.

NLR is an indicator of inflammation closely associated with tumor proliferation, invasion, and metastasis by affecting the tumor microenvironment (19, 20). Neutrophils participate in tumor initiation by producing reactive oxygen species(ROS), reactive nitrogen species (RNS), and proteases (21). Neutrophils recruited to tumor sites mainly promote cancer progression by increasing DNA damage, angiogenesis, and immunosuppression (22). Furthermore, neutrophil extracellular traps (NETs) are a new mechanism of cell death that has been shown to play a role in promoting the formation of tumor-associated thrombus and tumor progression (23). Lymphocytes also play an essential role in tumor immunity. Decreased lymphocytes decrease the immune response to tumor cells and increase the immune escape of tumor cells (5, 24). NLR has been widely used as an indicator for the differentiation of various benign and malignant tumors and for the poor prognosis of tumors (25). Viers et al. found that preoperative NLR values in RCC patients correlated with the pathological type of the tumor (26). Higher preoperative NLR in RCC patients might predict a more malignant histological subtype and larger tumor size. Unfortunately, this study did not include SRCC. Our study shows that higher NLR levels of SRCC compared to CCRCC are an independent risk factor for preoperative diagnosis. Higher NLR creates a worse tumor microenvironment, making the tumor more aggressive. Meng et al. found that the frequency of venous thrombosis and peritumor neovascularization was higher in SRCC compared to CCRCC (8). The possible reason was that at higher NLR levels, there are more inflammatory and immune cells in the tumor tissue, and these cells release more angiogenic factors in the hypoxic microenvironment (27).

We also evaluated the factors that may influence NLR. Using ROC to determine the cut-off values of continuous variables, we found that NLR levels in RCC patients correlated with age (P=0.040), gender(P=0.003), proteinuria(P=0.003), T stage(P=0.001), tumor size(P=0.013), TC(P<0.001), and TG(P<0.001). Further correlation analysis showed that NLR did not correlate with age(P=0.08) and TG(p=0.07) and only a weak correlation with tumor size(r=0.23) and TC(r=0.24). The results of subgroup analysis showed that SRCC was more likely in the high NLR group in all subgroups. The above results support that elevated NLR was a potential predictor for identifying SRCC.

Serum TC is an indicator of the patient’s caloric reserve and can reflect the patient’s nutritional status (28). The active metabolism of malignancy can cause cachexia and hypocholesterolemia (29). TC has been shown to be involved in the progression of RCC (30, 31), and the possible mechanism is the depletion of blood cholesterol by highly active LDL receptor-mediated endocytosis in cancer patients (32). In addition, lower cholesterol reduces monocytes’ antigen-presenting function and the number of circulating lymphocytes (28, 33). A multicenter study that included 3064 patients with RCC showed that preoperative TC levels were significantly lower in patients with SRCC (33). Our study showed that preoperative serum TC was lower in patients with SRCC compared to CCRCC, which may be due to the more aggressive nature of SRCC and faster tumor growth, resulting in a poorer nutritional status of patients. One report has shown that 23% of SRCC patients already had weight loss at the time of first diagnosis (17).

Previous studies have shown that tumor size and flank pain are associated with the diagnosis of SRCC (12, 13, 17). Approximately 90% of patients with SRCC are symptomatic at the time of presentation, with 52.3% presenting with flank pain at the time of first diagnosis. In comparison, more than 50% of all patients with CCRCC are asymptomatic (17, 34). These are consistent with our findings. In our research, patients with SRCC had larger tumor sizes and were more likely to present with flank pain. Compression by a larger tumor may be the main cause of flank pain.

Nomograms are already widely used in many cancers and are often more accurate in their predictive power than traditional methods. This study constructed a predictive model based on NLR, flank pain, tumor size, and TC for the preoperative differentiation between SRCC and CCRCC. As a predictive model incorporating 4 non-invasive indicators, it has a high clinical application in the preoperative diagnosis of SRCC. For example, a patient presented with flank pain and a large renal tumor. Hematological tests suggesting higher NLR and lower TC values indicate that the patient was at higher risk for SRCC. Using our predictive model (https://nomogramsrcc.shinyapps.io/DynNomappSrcc/), we could predict the probability of SRCC more accurately and provide patients with timely treatment recommendations. Notably, in the nomogram, we did not take cut-off values for continuous variables to make them categorical variables. Reaching a consensus on cut-off values for different study populations can be challenging. Using continuous variables can better score patients for risk. The results of the model’s ROC curves, calibration curves, and DCA curves supported the model’s good predictive power and clinical applicability. In addition, the data in our model are easily accessible and allow the timely identification of SRCC patients.

In this study, the included SRCC and CCRCC patients had tumor sizes > 4 cm. This was because when we collected case data from SRCC patients, we found only 2 cases of SRCC patients had sizes ≤ 4 cm. In order to make the predictive model more accurate, this exclusion criterion was eventually established.

This study also includes several limitations. (1) Retrospective studies were biased. We reduced this interference by setting strict exclusion criteria for inclusion. (2) Even though we randomly divided the data into training and control cohorts, the data came from a single center. (3) All patients in the study underwent partial or radical nephrectomy to ensure pathological accuracy and had tumors > 4 cm in size. Inevitably, there is a selection bias.

## Conclusion

In summary, this study suggested that NLR was a potential predictor for the preoperative identification of SRCC and CCRCC. The nomogram we constructed, including NLR, flank pain, tumor size, and TC, had excellent predictive ability and can provide clinicians with timely recommendations for identifying SRCC patients.

## Data availability statement

The raw data supporting the conclusions of this article will be made available by the authors, without undue reservation.

## Ethics statement

This retrospective study was approved by the Ethics Committee of Qilu Hospital of Shandong University. All participants signed informed consent forms, and all information was made anonymous.

## Author contributions

YW: project development, data collection, data analysis, figure design, manuscript writing and editing. NY: project development, figure design, manuscript writing and editing. TQ: data collection, figure design. XQ: data collection, figure design. ZZ: data collection, figure design. JZ: data analysis. QD: data analysis. The manuscript’s published form was approved by all authors. All authors contributed to the article and approved the submitted version.
