# Supplementary material for: A nomogram based on the preoperative neutrophil-to-lymphocyte ratio to distinguish sarcomatoid renal cell carcinoma from clear cell renal cell carcinoma
Source: Front Oncol. 2023 Sep 22;13:1218280. doi: 10.3389/fonc.2023.1218280 (PMC10556675; doi:10.3389/fonc.2023.1218280)
Supplement: Supplementary file 1 [file DataSheet_1.docx]

**Title*:* A nomogram based on the preoperative neutrophil-to-lymphocyte ratio to distinguish sarcomatoid renal cell carcinoma from clear cell renal cell carcinoma**

Yijian Wu^1,2^ , Tienan Qi^1,2^ , Xin Qin^1^, Zhongwei Zhao^1^ , Jianguo Zheng^1,2^ ,Qinglong Du^1,2^, Nengwang Yu^1*^

^1^ Department of Urology, Qilu Hospital of Shandong University, Jinan, Shandong, China;

^2^ Cheeloo College of Medicine, Shandong University, Jinan, Shandong, China.

**^*^Correspondence:**

Corresponding authors: Nengwang Yu, MD, PhD, Department of Urology, Qilu Hospital of Shandong University, Ji'nan, 250012, PR China. E-mail: [qiluyunengwang@hotmail.com](mailto:qiluyunengwang@hotmail.com);

**Supplement Table 1** Comparison of baseline characteristics for the training and validation cohorts

|  | Training(n=195) | Validation(n=85) | P-value |
| --- | --- | --- | --- |
| Age(years) |  |  | 0.130 |
| Mean(SD) | 56.4(10.8) | 58.5(10.0) |  |
| Median[Min,MAX] | 57[25,85] | 60[31,76] |  |
| Sex |  |  | 0.372 |
| Male | 127(65.1%) | 60(70.6%) |  |
| Female | 68(34.9%) | 25(29.4%) |  |
| Hypertension |  |  | 0.284 |
| Yes | 76(38.6%) | 25(29.4%) |  |
| No | 121(61.4%) | 60(70.6%) |  |
| Diabetes |  |  | 0.454 |
| Yes | 27(13.8%) | 9(10.6%) |  |
| No | 168(86.2%) | 76(89.4%) |  |
| Flank Pain |  |  | 0.569 |
| Yes | 43(21.8%) | 16(18.8%) |  |
| No | 154(78,2%) | 69(81.2%) |  |
| Haematuria |  |  | 0.182 |
| Yes | 29(14.7%) | 18(21.2%) |  |
| No | 168(85.3%) | 67(78.8%) |  |
| Proteinuria |  |  | 0.289 |
| Yes | 25(12.8%) | 15(17.6) |  |
| No | 170(87.2%) | 70(82.4%) |  |
| T stage |  |  | 0.627 |
| II | 152(77.9%) | 64(75.3%) |  |
| ≥III | 43(22.1%) | 21(24.7%) |  |
| Size(cm) |  |  | 0.202 |
| Mean(SD) | 6.5(2.0) | 6.8(2.2) |  |
| Median[Min,MAX] | 6.0[4.2,17.5] | 6.0[4.2,16] |  |
| White blood cell count(${10}^{9}$/L) |  |  | 0.723 |
| Mean(SD) | 6.30(1.62) | 6.36(1.98) |  |
| Median[Min,MAX] | 6.10[2.60,12.05] | 6.05[3.21,14.78] |  |
| Neutrophils count(${10}^{9}$/L) |  |  | 0.934 |
| Mean(SD) | 3.95(1.38) | 4.07(1.76) |  |
| Median[Min,MAX] | 3.86[0.96,9.17] | 3.77[1.45,12.87] |  |
| Lymphocyte count(${10}^{9}$/L) |  |  | 0.188 |
| Mean(SD) | 1.74(0.53) | 1.65(0.50) |  |
| Median[Min,MAX] | 1.67[0.53,3.36] | 1.54[0.75,2.88] |  |
| NLR |  |  | 0.197 |
| Mean(SD) | 2.51(1.45) | 2.76(1.94) |  |
| Median[Min,MAX] | 2.20[0.65,13.49] | 2.36[0.79,16.71] |  |
| TC(mmol/L) |  |  | 0.518 |
| Mean(SD) | 4.37(0.90) | 4.43(0.85) |  |
| Median[Min,MAX] | 4.37[1.03,6.75] | 4.40[2.31,6.75] |  |
| BUN(mmol/L) |  |  | 0.197 |
| Mean(SD) | 4.89(1.33) | 5.11(1.39) |  |
| Median[Min,MAX] | 4.79[2.30,9.80] | 4.98[2.67,8.93] |  |
| Cr(u mol) |  |  | 0.320 |
| Mean(SD) | 71(16.2) | 73(17.0) |  |
| Median[Min,MAX] | 68[38,129] | 70[38,126] |  |
| LDH(u/L) |  |  | 0.252 |
| Mean(SD) | 200(94) | 192(52) |  |
| Median[Min,MAX] | 187[79,1168] | 180[127,421] |  |
| TG(mmol/L) |  |  | 0.680 |
| Mean(SD) | 1.31(0.61) | 1.26(0.57) |  |
| Median[Min,MAX] | 1.15[0.32,3.62] | 1.12[0.38,2.93] |  |
| SRCC |  |  | 0.735 |
| Yes | 33(16.9%) | 13(15.3%) |  |
| No | 162(83.6%) | 72(84.7%) |  |

Abbreviations: NLR, neutrophil-to-lymphocyte ratio; TC, total cholesterol; BUN, blood urea nitrogen; Cr, creatinine; TG: triglycerides; SRCC, sarcomatoid renal cell carcinoma

Supplement Table 2 Univariate and multivariate logistic analyses for risk of SRCC in the training group

| Parameters | Univariate analysis  HR(95%CI) | P value | Multivariate analysis  HR(95%CI) | P value |
| --- | --- | --- | --- | --- |
| Flank Pain | 2.98(1.33~6.67) | 0.008 | 3.47(1.42~8.46) | 0.006 |
| Size | 1.35(1.14~1.61) | 0.001 | 1.29(1.07~1.56) | 0.007 |
| NLR | 1.41(1.09~1.81) | 0.008 | 1.28(1.01~1.62) | 0.044 |
| TC | 0.042(0.26~0.68) | 0.001 | 0.47(0.28~0.80) | 0.005 |

Supplementary Table 3 Comparison of the AUC of the nomogram with the AUC of the individual indicators in two cohorts

|  | Training cohort |  | Validation cohort |  |
| --- | --- | --- | --- | --- |
| Factors | AUC(95%CI) |  | AUC(95%CI) |  |
| Nomogram | 0.801(0.719~0.883) |  | 0.738(0.601~0.875) |  |
| NLR | 0.698(0.599~0.797) |  | 0.735(0.562~0.908) |  |
| Flank pain | 0.607(0.495~0.720) |  | 0.577(0.398~0.757) |  |
| TC | 0.725(0.630~0.820) |  | 0.558(0.404~0.712) |  |
| Tumor size | 0.684(0.583~0.784) |  | 0.697(0.538~0.856) |  |

Abbreviations: AUC, area under the curve; CI, confidence interval; NLR, neutrophil-to-lymphocyte ratio; TC, total cholesterol


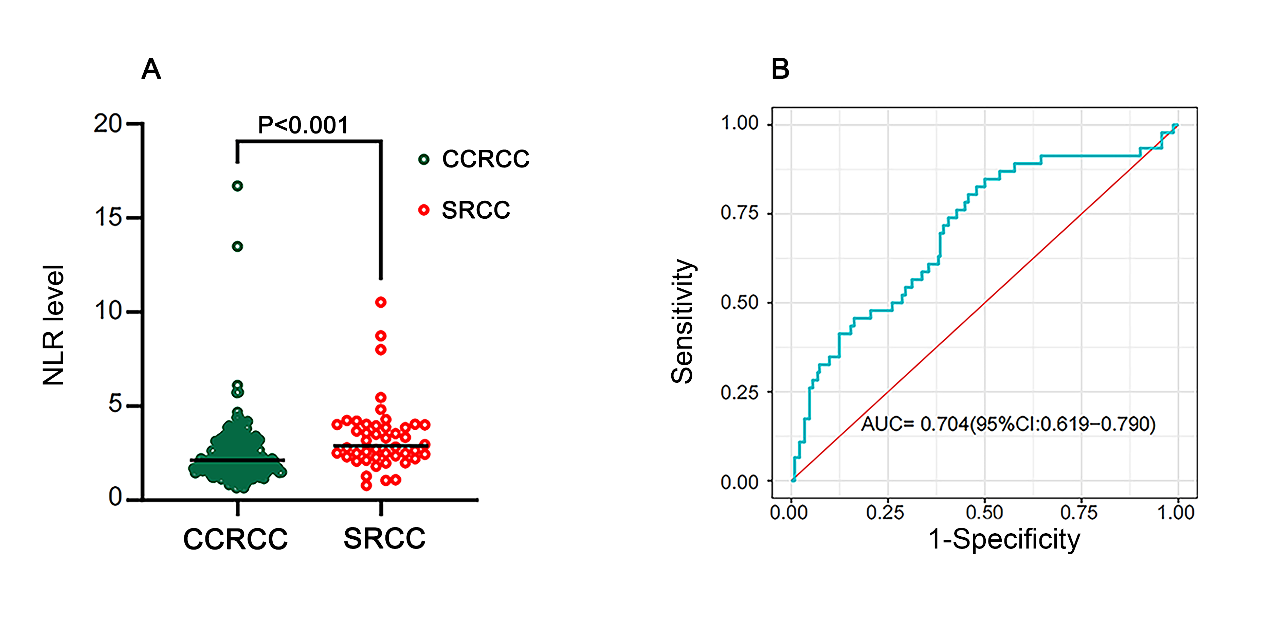


**Supplementary Figure 1.** (A) Comparison of NLR level between SRCC and CCRCC patients; (B) ROC curves of NLR in total patients. Abbreviations: NLR, neutrophil-to-lymphocyte ratio; CCRCC, clear cell renal cell carcinoma; SRCC, sarcomatoid renal cell carcinoma. ROC, receiver operating characteristic; AUC, area under the curve.


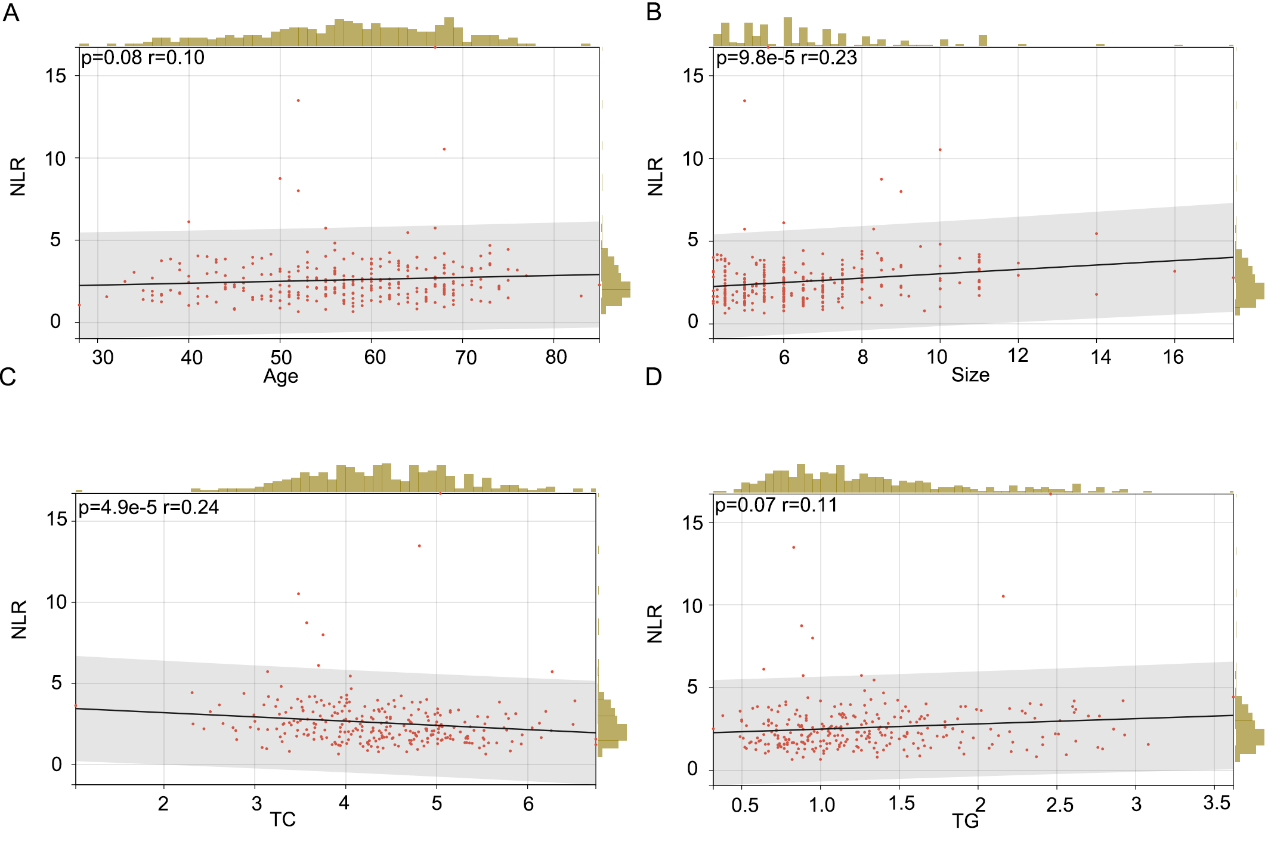


**Supplementary Figure 2.** Correlation analysis of NLR and age(A), size(B), TC(C), and TG(D). Abbreviations: NLR, neutrophil-to-lymphocyte ratio; TC, total cholesterol; TG: triglycerides.
